# England, Wales and Scotland Awakening
*Reprints of this number of "The Hospital" can be obtained on application to the Publisher, 28 & 29 Southampton Street, Strand, London, W.C.


**Published:** 1920-01-24

**Authors:** 


### ENGLAND, WALES AND SCOTLAND AWAKENING.*

Nearly every centre of population of any importance in England has exhibited inspiring evidence that the people are awakening to the call of humanity to help the Voluntary Hospitals. Birmingham, Bristol, Norwich, Leeds, and Bradford are additional centres which have shown signs of energy by the rapid development of active interest and in progressive movements towards co-ordination. Wales, through the Mayor of Cardiff, whose people never sleep when hospital needs call, has accomplished a record. The Mayor made an appeal for £150,000 for King Edward VII.'s Hospital there on Thursday, 15th instant, and by the 19th we had an announcement that the first donations received and promised exceeded £62,700. The largest contribution was one of £25,000 from Mr. Gould, M.P., and Mrs. Gould, and the smallest 2s. 9d. from J. G. Davison, Esq. This stimulating success, which we are confident can be equalled, if not surpassed, by many a large town in England and also in Scotland, is due to the confident note the Lord Mayor of Cardiff, Councillor J. F. Forsdike, M.P., adopted. Everywhere humanity claims both the money and the personal service of every thinking man and woman of intelligence throughout Great Britain to-day in support of the Voluntary Hospitals and their permanent establishment upon a sound financial basis. The great meeting at Cardiff impressed the observer by bringing the conviction that what the public wants, and especially shrewd business men, is to be assured and to know that they are asked everywhere to put their money down to support a well-managed and efficient organisation, tireless in overtaking the needs of the population, and insistent upon the most perfect administration of the hospitals for which the money is asked.

It is becoming manifestly plain that although all classes of the population are not insusceptible to sentiment, sentiment is not going to do much good unless it is supported by argument, and argument calls, between now and June 30, for the output of five months' energetic effort by the men and women, in every district, where there is a Voluntary Hospital, inviting their personal service, to bring the argument home to the people resident therein. Look at Cardiff and its Lord Mayor boldly asking for £150,000 for one hospital and receiving half of it in less than a hundred hours from his first utterance in support of his appeal. Cardiff has set the fashion. Everybody everywhere is anxious to be up and doing under an equally courageous and energetic Lord Mayor, or other local leader, wherever there is a Voluntary Hospital that needs financial help in large quantity, and deserves it. We are hopeful that some tens of thousands of Red Cross workers may become friendly workers in the cause of the hospitals, and we should like some Leader of a Red Cross organisation in every large town and active county throughout England, and the equivalent cities, towns, and counties throughout Scotland, to be up and doing, testing local feeling, and recruiting earnest men and women as friendly workers for the hospitals. Then when they have formed a company or regiment, to send us a postcard, naming their city or county where these battalions are organising, to be in readiness to set to work directly the Lord Mayor, Mayor, the Lord-Lieutenant, Colonel, the Lord Provost, or whoever may have the glorious privilege of leading this triumphant awakening, everywhere, of the people. They can, then, readily, save the hospitals, help their neighbours and themselves, and answer humanity's call with all their hearts and sympathies, energy, and brains. If this be done, as we feel confident it will be done, for it has already been begun, by June 30 next, if we all pull together and everywhere work for the success for the hospitals and the sick, the result cannot be doubtful, whilst the accomplishment will be magnificent.

### The Way to Hospital Victory.

The *South Wales News* of 16th inst. published a leading article entitled, "A Hospital's Appeal." It would be well worth while for every hospital supporter who is anxious to join the great army of workers and set about helping the hospitals in the right way, to obtain a copy of that paper, cut out the leading article, study and learn the drift of its main points, and then go forth and preach the gospel of humanity and succour for the Voluntary Hospitals, unceasingly, and with increasing force, until the great work is triumphantly brought to a successful conclusion, on June 30, 1920. What the workers have got to preach and to know is that the Voluntary Hospital does not only serve the needs of the active population in the town or city where it may be placed, but its activities far exceed these boundaries, and its succour, treatment, and nursing salvation are available to the whole of the industrial population in the mining valleys of some counties, in the agricultural districts of others, in the manufacturing centres throughout Great Britain, in the humblest cottage and in the wealthiest family circle within the reach of its succouring arms.

### The Twenty Millions who have never yet Helped a Hospital.

In such circumstances we must remember that there is at least one-half of the population who have the means, if they have the will, to help the hospitals (though they may never have helped them before), by joining the great army of workers and beginning to help to-day, wherever they may reside in the United Kingdom, and whoever they may be. It is Humanity's call to Help Humanity, and those who answer will thenceforward remember this great appeal for the Voluntary Hospitals and the sick.

*** Reprints of this number of "The Hospital" can be obtained on application to the Publisher, 28 & 29 Southampton Street, Strand, London, W.C.**

**England, Wales and Scotland AWAKENING**—(*cont.*).

Should they subsequently be ill themselves they will have earned the right to feel that, when health was theirs they remembered the sick, and God will remember them if their hour of trial comes. It is not necessary to-day to repeat the causes which have led to the enormous increase in the cost of treatment in hospitals since the days before the war. The fact of the need, which undoubtedly exists for the raising of substantial sums of money for hospital purposes, everywhere, throughout the United Kingdom, we have given already more than once. The Press, in London especially, and now we are grateful to notice that in the large towns too, the Press are giving space and brains to awaken the people to the actual facts, and so to attract them to the saving of the hospitals and the sick, throughout Great Britain. Then, too, though many charitable persons forget, or have never known, the truth, is, that the Voluntary Hospital has been badly hit by the war. No diligence or enforcement of economy could stay the growth of cost, because the expenses have been forced up by the general rise in prices, and **contributions have not kept pace with the increased expenses.**

### What a Local Press can Accomplish.

**So declares the "South Wales News," whose editor is not only a man of heart, but of knowledge, judgment, and apprehension. We want such a man in the midst of every population throughout the United Kingdom, aye, several men in every considerable centre of population, so that the local press may everywhere be joining hands with humanity to make the one central topic of conversation, the united effort of the hale and hearty man and woman, everywhere, to bring the provision for the succour and treatment of the sick up to a standard so high and far-extending, as to make their city an example of the true awakening of the people. There every man and woman will be proud to prove, that they are whole-heartedly doing their best, and doing it well and effectively. We all owe more than words can express to the efficient up-to-date Voluntary Hospital in our midst. The call is to our intelligence as well as to our humanity and heart, to keep up the standard by extending the area and volume of its work to an adequate point, and so providing, that in God's good providence, our city or our town, or even our village shall become famous for its healthy inhabitants, and the ever-diminishing number of the residents who, from any cause whatever, may need the succour of the hospital in a given year. Then our hospital, wherever it may be placed, will not wring the hearts of the passers-by by the crowds of persons to be seen always waiting outside its doors for admission, because their are no vacant beds to give them. Then the efficiency of its staff, and the economy of its management will bear favourable comparison with the very best in the land** Then, so long as there are any who require the hospital's services, so long will it be maintained at a pitch of completeness and financial strength to enable its managers to report continuously, that **never once for a single hour throughout a given year has there ever been a case waiting for admission, or an applicant qualified by his condition for hospital care remaining without it for a single minute from the time his incapacity was known and the sick patient arrived at the door of the voluntary hospital.**

### A Memorable Meeting.

On Friday, 23rd inst., an important meeting of the representatives and managers of the principal hospitals throughout Great Britain is to be held in the Great Hall of St. Thomas's Hospital, to take counsel together as to the financial needs and requirements of our Voluntary Hospitals, to hear the Hon. Sir Arthur Stanley's tender of help on behalf of the Red Cross, to consider the scheme which Sir Henry Burdett put forward in the *Times* of December 17, which appeared in The Hospital of December 20, 1919, on pages 268 and 269. They should further hear suggestions, receive tenders of help, and gather, as we hope, many points of interest which will send our friends, from all parts of the United Kingdom, back home, with the inspiration and the material to enable every one of them to demonstrate how to organise their great appeal at home, and to recruit successfully the majority, at any rate, of **half the adult members of the population, which probably number one-half of the total population, who have never yet given anything or done anything materially to help the hospitals. These people may now enjoy the privilege of awakening to the call of humanity, may set themselves with intelligent and joyous hearts to deliberately give each year a portion of their time to the congenial and self-imposed task of friendly workers in the days of health in the cause of the sick and injured. By such voluntary service they may bring to the great Voluntary Hospitals which afford their patients succour, the best means of restoration to renewed health and remunerative activi'y.**

### The Ball is Rolling, the Time Opportune, and Now for the Reapers.

The ball has been set a-rolling, the time is opportune, the opportunity big enough and all-embracing enough to fire the imagination of the dullest of mortals, and we make no doubt that the latent powers resting in each community, which is at present concentrating in the quiet men and women who have always been retiring, but who possess, as we have discovered in many places in the past, splendid energy in reserve, if only they are given an opportunity which appeals to them, to enter into the privileges which attach to all friendly workers among the sick, who have been awakened to their opportunities and the joys which they bring by the fulfilment of the energetic action, to which the Great Giver of all good has invited men and women from time immemorial.

What Every Population Needs.

The marvels Cardiff has accomplished in less than one week at least 250 other centres of population can do to an equal or even greater extent. All that is required is the leadership of a few energetic people, and the co-operation with them of many hundreds of the resident population of all classes who will form The Hospital Company, or Regiment, or Brigade, according to their numbers. These, when charged with the self-imposed privileges, will secure by united effort the additional financial aid and other resources, the absence of which, at the moment, are so threatening to the efficient upkeep and adequate resources of the Voluntary Hospital, as to imperil its very existence. This pressing crisis can, however, and will, however, be readily and speedily ended, wherever those who heretofore gave little or nothing to the Voluntary Hospitals, awaken from their slumbers, study and satisfy themselves as to the facts, and then turn to as Friendly Workers and Reapers of the Harvest Field. This has been demonstrably proved, in many ways in the past, to be practically illimitable in resources, providing only the Claims and Needs **are brought home to the individual.** Then not only the means, the money, and the resources will flow in, and continue, but all who become friendly workers and intelligent supporters of the Voluntary Hospital will become alive to the realities of existence, and so come to thank God and take courage, whilst they rejoice in the privileges personal service, in the days of Health in the cause of the sick, has brought to them, as the supreme fruit of all their self-denying methods.

